# Wild Rodent Ectoparasites Collected from Northwestern Iran

**Published:** 2017-03-14

**Authors:** Zabihollah Zarei, Mehdi Mohebali, Zahra Heidari, Eshrat Beigom Kia, Amrollah Azarm, Hasan Bakhshi, Jaber Davoodi, Hamid Hassanpour, Manizhe Roohnavaz, Mahya Khodabakhsh, Zakkyeh Telmadarraiy

**Affiliations:** 1 Department of Medical Parasitology and Mycology, School of Public Health, Tehran University of Medical Sciences, Tehran, Iran; 2 Centers for Research of Endemic Parasites of Iran (CREPI), Tehran University of Medical Sciences, Tehran, Iran; 3 Department of Medical Entomology and Vector Control, Faculty of Medical Sciences, University of Tarbiat Modarres, Tehran, Iran; 4 Malaria and Vector Research Group, Biotechnology Research Center, Pasteur Institute of Iran, Tehran, Iran; 5 Department of Veterinary Parasitology, Islamic Azad University Abhar Branch, Abhar, Iran; 6 Department of Veterinary Internal Diseases, Faculty of Veterinary Medicine, Tehran University, Tehran, Iran; 7 Department of Medical Entomology and Vector Control, Tehran University of Medical Sciences, Tehran, Iran

**Keywords:** Rodent, Ectoparasites, Iran

## Abstract

**Background:**

Rodents play an important role as reservoir of some pathogens, and the host of some ectoparasites as well. These ectoparasites can transmit rodents’ pathogens to human or animals. The aim of this study was to assess the distribution and infestation load of ectoparasites on rodents in Meshkin-Shahr District, northwestern Iran.

**Methods:**

Rodents were captured using baited live traps in spring 2014 from Meshkin-Shahr District and were transferred to the laboratory for identification to the species level. Their ectoparasites were collected, mounted and identified.

**Results:**

Three rodent species including *Meriones persicus* (74%), *Mus musculus* (16.9%) and *Cricetulus migratorius* (9%) were identified. Among all rodents, 185 specimens (90.69%) were infested with a total of 521 ectoparasites. Overall, 10 arthropods species were collected, including fleas (97.6%), one mite (1.6%) and one louse species (0.6%) as follows: *Xenopsylla nubica*, *X. astia*, *X. buxtoni*, *X. cheopis*, *Nosopsyllus fasciatus*, *N. iranus*, *Ctenocephalides felis*, *Ctenophthalmus rettigismiti*, *Ornithonyssus* sp and one species of genus *Polyplax*. The most prevalent ectoparasites species was *X. nubica* (89%).

**Conclusion:**

Nearly all rodent species were infested with *Xenopsylla* species. Monitoring of ectoparasites on infested rodents is very important for awareness and early warning towards control of arthropod-borne diseases.

## Introduction

Rodents play important roles in disease transmission via their urine, feces, bite and transmission of pathogenic agents through ectoparasites (Williams et al. 1997). Some viral, bacterial and protozoal agents as well as helminthes resulting in leishmaniasis, Crimean-Congo Haemorrhagic Fever (CCHF), plague, leptospirosis, salmonellosis, rat-bite fever, Omsk hemorrhagic fever, anaplasmosis, ehrlichiosis, murine typhus, theileriosis and babesiosis can be transmitted by rodents to other animals orhumans when they are in close contact (Williams et al. 1997, Tsuji et al. 2001, Motevalli-Hagghi et al. 2002, Telmadarraiy et al. 2007, Kia et al. 2009, Tajedin et al. 2009, Eisen and Gage 2012, Nateghpour et al. 2013). Fleas (Siphonaptera) and Ixodid ticks (Acari: Ixodidae) are the major vectors of important pathogens threatening human and animals (Goz et al. 2016).

Some ectoparasites of medical and veterinary importance were found and reported from rodents in different parts of Iran (Mohebali et al. 1997, Kia et al. 2001, Telmadarraiy et al. 2004, Shayan and Rafinejad 2006, Hanafi-Bojd et al. 2007, Telmadarraiy et al. 2007, Sharifi et al. 2008, Shoorijeh et al. 2008, Kia et al. 2009, Tajedin et al. 2009, Nateghpour et al. 2013, Zendehfili et al. 2014, Telmadarraiy et al. 2015). In those studies some rodents species such as *Meriones lybicus*, *M. persicus*, *M. hurrianae*, *Tateraindica*, *Mus musculus*, *Rattus rattus*, *R. norvegicus*, *Nesokia indica*, *Microtus socialis*, *Gerbillus nanus*, *Glis glis*, *Apodemus sylvaticus* and soon were studies and the ectoparasite species were included *Pulex irritans*, *Xenopsylla cheopis*, *X. astia*, *X. buxtoni*, *X. nubica*, *X. conformis*, *Nosopsyllus medus*, *N. fasciatus*, *Polyplaxs pinulosa*, *P. gerbilli*, *Rhipicephalus* sp, *Hyalomma* sp, *Boophilus* sp*, Laelaps nuttalli*, *L. ciccuminata,* And *rolaelaps hermaphrodita*, *Paracheylaelaps pyriformis*, *Dermanysus sanguineus*, *D. americanus*, *Ornithonyssus bacoti*, *Haplopleura captiosa*, *Haemolaelaps glasgowi*, *Echinolaelaps echidninus* and some other species.

Because of high climatic diversity in Iran and numerous ecological niches of rodents, there is a considerable potential for survey on their ectoparasites to find the vulnerability of vector-borne zoonoses in the country.

There was no comprehensive study on the rodent and their ectoparasites in northwest of Iran. Therefore, the aim of this study was to assess the distribution and infestation level of ectoparasites on rodents in selected sites in Meshkin-Shahr County located in northwest of the country. This will help us in monitoring ectoparasites infestation to alerting and early warning to possibility for control of arthropod-borne diseases in northwestern part of Iran.

## Materials and Methods

### Study area

**Fig 1:**
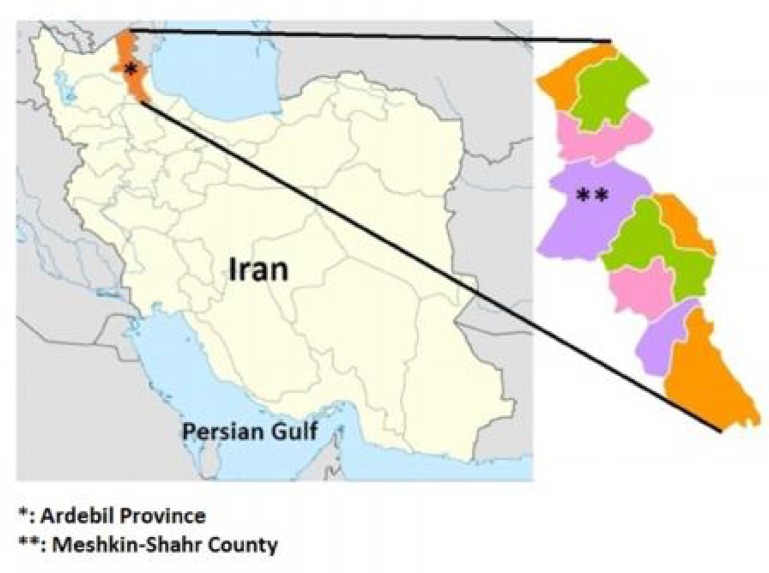
Location of Ardebil Province. This province is located in Northwest part of Iran and is bordered with Republic of Azerbaijan. Meshkin-Shahr County is one of the counties of the province.

Meshkin-Shahr County (38° 26′N 47° 45′E) is one of the counties located in Ardebil Province (Fig. 1). The province is considered the coldest province in Iran. Large parts of the province are forested and green. Neighboring the Caspian Sea and the Republic of Azerbaijan, the province is of great economic significance as well as splendid natural beauty and numerous sights. Meshkin-Shahr County is located at an elevation of 1,341 meters above sea level, the whole district occupying the foot-hills of Sabalan Mountain. The weather is extremely cold in the winter (down to −27 °C) and warm during the summer (up to 40 °C). Many tourists come to the region for its cool climate.

### Rodent collection

Rodents were live trapped at different localities including Altisiluo, Khiav, Abiz, Our, Oudkandi and Magandeh. Rodent’s collection was carried out on various occasions and different places like indoor places, farms, roadsides and other places in spring 2014. Live traps were randomly set in different aforementioned habitats baiting with favorable food of rodents according to the season. These traps were set at different parts of selected regions of the district from 11:00 AM to the next day.

### Ectoparasites collection

Captured rodents were transported rapidly to the laboratory of Meshkin-Shahr Research Station and their ectoparasites were isolated using brushing on the fur or by a fine forceps immediately after transporting. Collected ectoparasites were stored in 70% ethanol for preservation and identification.

### Rodents and ectoparasites identification

Different morphological criteria were used for identification of rodents in the species level by preparation of rodent skulls, mounting of ectoparasites, identification of rodents (Etemad 1976) and ectoparasites (Strandtmann and Wharton 1958). Ectoparasites specimens were fixed in between microscope slides and cover glass and confirmation of some species was carried out according to standard methods provided.

## Results

**Fig 2:**
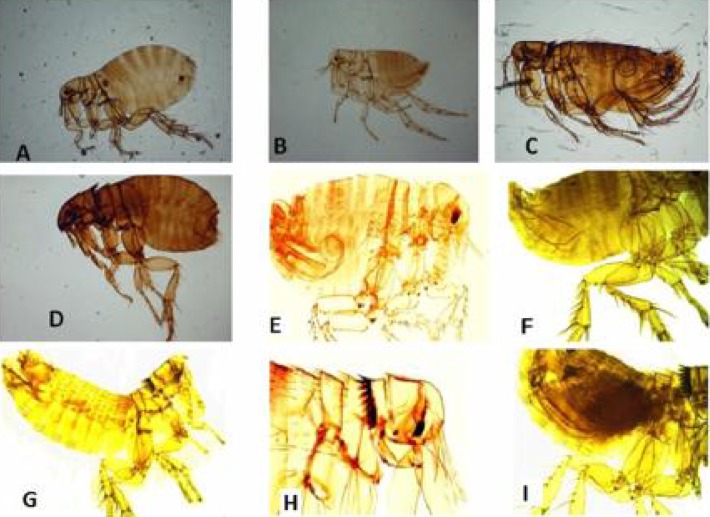
Some collected ectoparasites; A: *Xenopsylla nubica*, B: *X. nubica*, C: *Nosopsyllus fasciatus*, D: *Ctenocephalides felis*, E: *X. cheopis*, F: *X. astia*, G: *Ctenophthalmus rettigismiti*, H and I: *N. iranus*

A total of 204 rodents were identified in 3 species including *Meriones persicus* (74%), *Mus musculus* (16.9%), and *Cricetulus migratorius* (9%). Among them, 185 (90.69%) were infested with 521 ectoparasites. The ectoparasites of the rodents were comprised mites, fleas and lice (Fig. 2).

Totally, 10 species were captured in this study including eight fleas (97.6%), one mite (1.6%) and one species of genus *Polyplax* (0.6%) species as follows: *X. nubica*, *X. astia*, *X. buxtoni*, *X. cheopis*, *Nosopsyllus fasciatus*, *N. iranus*, *Ctenocephalides felis*, *Ctenophthalmus rettigismiti*, *Ornithonyssus* sp, and *Polyplax* sp (Table 1).

**Table 1.** Details of collected ectoparasites from caught rodents. These ectoparasites were collected in spring 2014 from Meshkin-Shahr District located in northwest part of Iran

**Ectoparasites**	**Male**	**Female**	**Total**	**Total (%)**
***Xenopsylla nubica***	331	134	465	89
***X. astia***	8	3	11	2
***Nosopsyllus fasciatus***	5	7	12	2.4
***Xenopsylla buxtoni***	1	1	2	0.4
***Xenopsylla cheopis***	8	2	10	1.9
***Ctenocephalides felis***	1	2	3	0.6
***Nosopsyllus iranus***	1	3	4	0.9
***Ctenophthalmus rettigi smiti***	1	2	3	0.6
***Ornithonyssus* sp**	5	3	8	1.6
***Polyplax* sp**	2	1	3	0.6

## Discussion

In the present investigation, three rodent species including *Meriones persicus*, *Mus musculus* and *Cricetulus migratorius* were caught in Meshkin-Shahr District located in Ardebil Province, northwest of Iran during spring 2014.

Study on these rodents is essential as they have an important role as host of many parasitic agents (Darvish et al. 2015). Out of 204 collected rodents, *M. persicus* was the most prevalent species in that area (74%). In the former investigations, occurrence of this rodent has been revealed in Central Asia, Transcaucasia, Turkey and Pakistan (Carleton and Musser 2005). Moreover, in northwest of Iran, this species was found in Maku and Urumiyeh located in West Azerbaijan Province as well as Jolfa located in East Azerbaijan Province (Lay 1967). In a recent investigation in northwest part of Iran, this species has been found in Kordasht, Sufian and Tabriz (East Azerbaijan Province) as well Zanjan County in Zanjan Province (Darvish et al. 2015). This species is introduced as a probable reservoir for visceral leishmaniasis in Iran (Shojaei and Mohebali 2005). The presence of this species in Meshkin-Shahr District is consistent with the above mentioned investigations. Furthermore, we could find *Mus musculus* in our investigation. This species has been found in almost all parts of the country (Etemad 1976). Another species *C. migratorius*, has been captured in Meshkin-Shahr and reported with high titers of *Leishmania* antibody in Meshkin-Shahr (Mohebali et al. 1995, 1997).

Ectoparasites play an important role in transmitting some diseases to human and domestic animals (Williams et al. 1997, Eisen and Gage 2012). Therefore, study on ectoparasites will help investigators to evaluate the possibility of pathogens transmission in a given area.

In this study, the most prevalent (89%) ectoparasite was *X. nubica*. This species has been introduced as the vector of plague in Mauritania (Eisen and Gage 2012). Among other flea species collected in this study *X. astia*, *X. buxtoni*, *X. cheopis* and *N. fasciatus* are introduced as the vectors of plague in Southeast Asia and the Western Pacific, Iran, Libya, Mauritania, East Africa, Southern Africa and South America (Eisen and Gage 2012).

In Meshkin-Shahr County, *M. persicus* was the dominant rodent species, while 7 species of ectoparasites were identified as: *X. gerbilli*, *X. nuttalli*, *N. fasciatus*, *Stenopnia tripectinata*, *Ornitonyssus bacoti*, *Trichoecius romboutsi* and *Haemaphysalis* sp. (Mohebali et al. 1997). Comparing with their study we could not capture *Alactage elater* rodent species, while the common ectoparasite in both studies is *N. fasciatus*.

In another study in Germi, Ardebil Province, two species of rodents were trapped: *M. persicus* and *Microtus socialis* with the frequency of 90.4% and 9.6%, respectively (Kia et al. 2010). The frequency of *M. persicus* is in concordance with our result. Although we did not find *M. socialis* in the present investigation, but two other species, i.e., *C. migratorius* and *M. musculus* species were trapped in our study.

Other investigations carried out in west of Iran reported some ectoparasite including *Pulex irritans*, *X. buxtoni*, *Nosopsyllus medus*, *Polyplax spinolosa*, *Rhipicephalus* sp, *Hyalomma* sp, *Lealaps nuttalli*, *Dermanysus sanguineus* and *Ornithonussus bacoti* (Telmadarraiy et al. 2007). The difference between this study with our investigation may be due to the host and climate. The importance of ectoparasites has encouraged investigators to study on other animals like hedgehogs (Goz et al. 2016). In a most recent investigation carried out on 21 hedgehogs ectoparasites in east part of Turkey (northwest of Iran), *R. turanicus* and *A. erinacei* were detected, also, infestation rate for ticks and fleas was detected as 66.6% and 100%, respectively (Goz et al. 2016). We could not collect these ectoparasites on the caught rodents. This is expected to be so, because *A. erinacei* is the ectoparasite of hedgehogs, cats and dogs (Pomycal 1985).

## Conclusion

Five flea species identified in this study are introduced as the vectors of plague in different foci of the disease around the world, so it is recommended to do a serological study for plague in rodents and their ectoparasite in this area. Monitoring of ectoparasites on the infested rodents is very important for awareness and early warning towards control of arthropod-borne diseases.
